# Retrieval Practice for Improving Long-Term Retention in Anatomical Education: A Quasi-Experimental Study

**DOI:** 10.1007/s40670-021-01298-8

**Published:** 2021-05-11

**Authors:** Mohammad B. Azzam, Ronald A. Easteal

**Affiliations:** 1 Faculty of Education, Western University, London, ON N6G 1G7 Canada; 2 Department of Biomedical and Molecular Sciences, Queen’s University, Kingston, ON K7L 3N6 Canada

**Keywords:** Memory, Working memory, Retrieval, Retrieval practice, Learning

## Abstract

It is generally assumed by students that learning takes place during repeated episodes of rereading and rote memorization of course materials. Over the past few decades, however, research has increasingly indicated that the said notion can and should be enhanced with learning paradigms such as retrieval practice (RP). RP occurs when students practice retrieving their consolidated semantic memories by informally testing themselves. This strategy results in the re-encoding and re-consolidation of existing semantic memories, thus strengthening their schemas. The purpose of this quasi-experimental design was to assess the effects of the implementation of RP on student performance on the final exam in a large, undergraduate Gross Anatomy course. It was hypothesized that student participation in RP during class would improve their performance on the final exam in the course. The participants (*N* = 248) were mainly in Life Sciences, Kinesiology, and Physical Education programs. They answered RP questions using TopHat©, an online educational software platform. The results of this study indicated that student performance on the final exam was enhanced when students engaged in RP. It was concluded that the use of RP effectively enhances learning and long-term retention of semantic memory. In addition to the traditional testing ‘of’ learning, teachers are encouraged to implement testing, in the form of RP, in their classrooms ‘for’ learning.

## Introduction

Learning of new, semantic (factual) information is traditionally thought by students to typically occur during episodes of rote memorization [1]. Historically, this notion has been, and in many cases still is, emphasized in scientific disciplines such as anatomy. Recent achievements in the research on learning have indicated that if teachers and students take advantage of how semantic memory and its processes function, learning can be more effective. One learning paradigm, which utilizes semantic memory, is retrieval practice. Retrieval practice (RP) refers to the use of testing as a form of learning and has been shown to be effective [2–7]. This study examined the use of RP and its effectiveness in a large, undergraduate Gross Anatomy course at a Canadian university. This paper argues that testing in the form of RP should be used as a method of learning.

### Memory: Stages and Processes

Semantic memory, or that of factual information, can be classified as one of four stages, where the transfer from one stage to the next is completed by one of three processes. The first of these stages is sensory memory (SM) [8], where the information perceived by vision and audition [9] is stored as a memory within the visual and auditory cortices for approximately 2 s [10]. SM is then transferred into working memory (WM), which is temporarily held in the dorsolateral prefrontal cortex (DL-PFC). Like SM, WM is also limited in both capacity and duration and can hold an average of five to seven items at once, for up to 12 s [11]. It is within the WM that information can be manipulated (e.g., strengthened or weakened); thus, it is said that a memory in WM is in its labile state [12].

Memory from the WM is transferred to both the hippocampus (HPC) as recent memory (RM) and the association cortex as long-term memory (LTM) simultaneously. This process—the initial registration of information—is known as encoding [13]. Encoding creates rather weak representations, or schemas, of the memory. RM can be stored in the HPC for approximately 5 days [14], and is then transferred into LTM. Unlike other stages of memory, memory in LTM is in its permanent state and can be stored indefinitely. This process—whereby memory is stabilized from its labile state into its permanent state—is known as consolidation [13]. A semantic memory is consolidated by the process of long-term potentiation (LTP), by which its processing in the HPC results in increased synaptic strength [15]. Lastly, the ability to access memory from LTM, return it to WM, and use it is known as retrieval [13]. The repeated consolidation and retrieval of a memory strengthens the schemas that are associated with that memory [3]. Moreover, the stronger the schemas are, the more easily the information is remembered.

### Implementing Retrieval Practice

Hence, taking advantage of the described neuroanatomical processes in the form of RP is especially important in education. Researchers [e.g., 2–7] over the past few decades have demonstrated that RP is a superior mnemonic device, compared to repeated rote memorization on its own, at enhancing the long-term retention of semantic memory. Further, the effectiveness of RP can be especially useful in Higher Education, and even more so in Science Education [16], as factual information is constantly delivered and is expected to be learnt and remembered accurately. For instance, Dobson et al. [17] had conducted a week-long study of RP in learning skeletal muscle anatomy with 72 Kinesiology students to demonstrate that RP strategies combined with spaced learning generated improved recall of information, compared to memorization and massed learning. Further, Larsen et al. [18] had conducted a study in the natural settings of medical resident learning of myasthenia gravis and epilepticus. Participants were randomly divided into two groups, each responsible for reviewing one neurological disorder and then completing a test on the other. A test on both neurological disorders 6 months later revealed that RP resulted in test scores being 13% higher compared to the study condition.

It is imperative to point out that the provision of feedback when implementing RP is particularly important [19], especially when RP is implemented using recognition tests (e.g., multiple-choice questions). This is because students may encode incorrect information as correct [20], and therefore may consolidate and then reproduce the wrong answers on subsequent assessments [21]. Further, perhaps the greatest challenge towards the successful implementation of RP is that most students do not believe that it works [1]. This may be explained by the student’s metacognitive awareness, or the lack thereof, of the testing and retrieval effects [22]. Therefore, teachers are encouraged to provide research-informed evidence to their students to convince them of the efficacy of RP before attempting to use it in their classrooms.

### The Present Study

This study employed a quasi-experimental design in a large, undergraduate Gross Anatomy course that was designed for students who wanted to learn about the human body’s visceral systems. This study intended to address the question, “Does student participation in RP during class improve their performance on the final exam in the course?”

## Materials and Methods

This quasi-experimental study examined the effectiveness of RP on student learning, and in turn, their performance, on the final exam in a large, undergraduate Gross Anatomy course at Queen’s University, Kingston, ON, Canada. The course ran throughout the semester for 12 weeks, with three lectures and a 2-h lab in the Anatomy Learning Centre per week. The course discussed the gross anatomical structures in the head, neck, and thorax, including detailed descriptions of the structures’ neurovasculature. The instructor typically employs instructional practices such as the use of the course textbook and the document camera to teach this course, where they highlight important textual information and label figures and images, as well as present anatomical specimens and models obtained from the Anatomy Learning Centre. In past years, when RP was not practiced in this class, the student average was between 65 and 70%. This study was approved by the Health Sciences and Affiliated Teaching Hospitals Research Ethics Board (HSREB) at Queen’s University.

### Participants

All participants (*N* = 248) in this study were full-time students who were enrolled in a large, undergraduate Gross Anatomy course. For most students, this course was a requirement for credits towards their respective degrees, and therefore, most of them regularly attended all lectures. These students were enrolled in one of three programs: third-year Life Sciences (LISC) (*N* = 60), second-year Kinesiology (KIN) (*N* = 121), and third-year Physical Education (PHED) (*N* = 43). All other students, collectively classified as ‘Others’ (*N* = 24), enrolled in this course as an elective. Participation in this research was completely voluntary and participants had to provide informed consent.

### Procedure

All enrolled students participated in this study. Participation was completed on TopHat©, an online educational, non-analytical software platform that students were able to download as an app on their electronic devices, or access online via https://tophat.com/. The use of TopHat©, unlike other methods such as the use of clickers, allowed for the application of delayed retrieval—a fundamental strategy of RP which makes retrieval effortful [23]. Student usage data were collected from TopHat© servers and de-identified. All analysis was done using these de-identified data sets. Students were given grades for their participation, up to a maximum of 5% of their overall course grade.

RP was implemented during the first 10 min of each lecture, whereby 6 to 10 questions were posed. Equal interval spacing was used during this study, whereby students were stimulated to practice retrieval of semantic information, comprehension, and application in equal temporal intervals. This strategy worked well in this course as the instructor had employed the Spiral Syllabus [24], where new information would build upon previous information in a logical, sequential manner. The most important concept areas in this course, and therefore the most repetitive RP questions, were concerned with the neurovasculature of the respective gross anatomical structures. Feedback in the form of correct answers was given to students 1 day after each RP session. The final exam was written approximately 3 weeks after the course ended.

All the questions posed during the course, in both RP and on the final exam, were multiple-choice questions (recognition tests). A total of 252 RP questions were posed during the semester and a total of 148 questions were posed on the final exam. RP was used to study student knowledge of the innervation of the visceral systems; thus, of all the questions that were asked on the final exam, only the questions that addressed innervation were congruent with the questions posed during RP. These questions, which amounted to a total of 67 final exam questions, were designated as completely congruent questions (CCQs).

‘Congruency’ refers to the degree that a RP question is similar to a question that was posed on the final exam. This does not imply that the same question was asked during both the implementation of RP and on the final exam; rather, two questions were considered congruent if the same knowledge that pertains to one also pertains to the other. For example, “Which of the following nerves innervates the masseter muscle?” was asked as an RP question; a CCQ congruent with this question was “Which cranial nerve, if damaged, causes inability to chew?”

All other questions, which did not address innervation and were posed during RP but not on the final exam, were designated as noncongruent questions (NCQs). An example of an NCQ was “Which of the following is not a bone of the face?” Of course, students were not made aware of these categorizations and were expected to study and learn all the information that was taught during the course.

### Data Analysis

The students’ overall levels of participation in RP were obtained from the tabulated statistics on TopHat©. Furthermore, their grades for each of the CCQs were obtained from TopHat© and their final exam answer sheets. All data were tabulated, and data analysis was performed according to demographics. Students were categorized into one of two groups based on the level of their participation. Participation was characterized as the total number of RP questions the students answered during the semester, regardless of the correctness of those answers. The dichotomous cut-off for participation was set at 85% due to a natural gap in the data (see Table 1), as determined by a consulting statistician in the faculty. Therefore, students who participated ≥ 85% were classified as the high RP group, and those who participated < 85% were classified as the low RP group. The participants in both groups were similar in terms of intellectual ability, as measured by their GPA prior to the commencement of the course.

**Table 1:** Student participation in retrieval practice (RP), according to demographics

Program	Participation			Total
	*x* ≤ 80%	80% < *x* ≤ 90%	*x* > 90%	
Life Sciences	6	9	45	60
Kinesiology	40	18	63	121
Physical Education	21	5	17	43
Others	10	0	14	24
Total	77	32	139	248

The Statistical Package for the Social Sciences (SPSS Statistics) was used to analyze the data. Statistical significance was set at *p* < 0.05. In all statistical analyses, the dependent variable was always student performance on the final exam; the independent variable was student participation on TopHat©. A Shapiro-Wilk’s test was conducted to test for normality of both dependent and independent variables. This indicated that the data were non-normally distributed. Kendall’s tau-b and Spearman’s rank-order correlations, as well as linear regression, were performed to determine the existence (or lack thereof) and strength of the relationship between the dependent and independent variables.

## Results

A total of 248 students participated in this study, 200 of whom participated ≥ 85% of the time; thus, they were designated as the high RP group. The remainder (*N* = 48) were designated as the low RP group. All participants had participated to some degree throughout the semester. Table 2 summarizes the demographics of each group.

**Table 2:** Student participation in retrieval practice (RP), according to demographics

Program	Designated group		Total
	High RP group	Low RP group	
Life Sciences	55	5	60
	27.5%	10.4%	24.2%
Kinesiology	98	23	121
	49.0%	48.0%	48.8%
Physical Education	33	10	43
	16.5%	20.8%	17.3%
Others	14	10	24
	7.0%	20.8%	9.7%
Total	200	48	248
	100%	100%	100%

Percentages are calculated by dividing the number of group participants enrolled in each educational program by the total number of participants in that group. For example, 27.5%, or 55 out of 200 high RP participants, were enrolled in Life Sciences

The average grade for all CCQs in RP during class for the high RP group was 91.6% (*SD* = 1.6%), while their average grade for all CCQs on the final exam was 75.8% (*SD* = 3.7%). Similarly, the average grades for the low RP group were 73.5% (*SD* = 2.1%) and 62.3% (*SD* = 1.6%), respectively. These results indicated that the high RP group performed better on the CCQs on the final exam, relative to the low RP group.

**Fig. 1:**
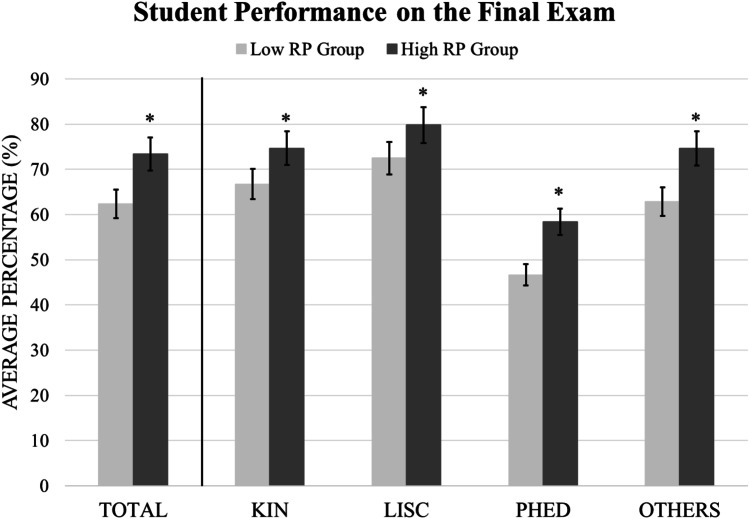
Average scores on the final exam according to demographics. Asterisks represent significance (*p* < .05). Error bars represent standard deviation (SD). Notes: KIN, Kinesiology; LISC, Life Sciences; PHED, Physical Education; RP, retrieval practice

When the data were analyzed according to demographics, it was demonstrated that the high RP groups performed better on their final exams compared to the low RP groups: KIN (*p* < 0.001), LISC (*p* = 0.031), PHED (*p* = 0.046), and Others (*p* < 0.001). These results are shown in Fig. 1.

A Shapiro-Wilk’s test (*p* > 0.05) was performed to test for normality, which illustrated that the variables were non-normally distributed. Kendall tau-b and Spearman’s rank-order correlations were performed to assess the relationship between student participation in RP and student performance on the final exam. There were moderate, positive correlations between student participation in RP and student performance on the final exam, which were statistically significant, *τ*_b_ = 0.334, *p* < 0.001, and *r*_s_ = 0.420, *p* < 0.001.

Linear regression was run to understand the effects of the dichotomized student participation (set at 85.00%) in RP on student performance on the final exam. It was demonstrated that student participation on the CCQs in RP statistically significantly predicted their performances on the CCQs on the final exam, *F*(2, 245) = 54.62, *p* < 0.001, accounting for 30.80% of the variation in student performance with an adjusted *R*^2^ = 30.30%.

## Discussion

The purpose of this study was to understand the effectiveness of classroom-based RP on student performance on formal evaluations in a large, undergraduate Gross Anatomy course. RP was implemented throughout the semester. The CCQs during RP specifically addressed the innervation of the visceral systems of the head, neck, and torso.

Data analyses demonstrated that the utilization of RP in the classroom was statistically significantly associated with improved student performance on formal evaluations. More particularly, students who were enrolled in PHED as well as those who took the course as an elective benefited the most from the implementation of RP, compared to the LISC and KIN students. In addition, it was demonstrated that the LISC students in both low and high RP groups performed better than the students from other programs. A possible explanation for these findings may be that the students who participated more frequently were more conscientious, and that conscientiousness may be the actual driving factor for increased final exam performance. Another explanation may be that the LISC students, compared to the KIN students, were upper-year students who were generally looking to applying to professional schools—especially medicine. Nonetheless, the implementation of RP was associated with improved student performance on the final exam, irrespective of program. The implications of this research illustrate that implementing RP enhances learning experiences and retention of semantic information in Anatomical Education. Although these findings are especially significant in disciplines such as anatomy, RP can successfully be implemented in any other discipline.

### Limitations

Perhaps one of the greatest limitations was that students may have engaged in peer instructional pedagogical techniques when attempting to answer RP questions, despite being repeatedly discouraged from so doing. If a group of students answered RP questions together, it is plausible that only one of them may have successfully retrieved the memory from LTM, while the others may have merely recognized that the answer was correct.

In addition, the implementation of a randomized experimental design under strict, laboratory-controlled conditions would have been unethical because students in the control group would have been put at a disadvantage due to the positive effects of RP on the experimental group. This made it difficult to account for several factors, such as previous knowledge and level of interest in anatomy, that may have influenced the results of this study.

Lastly, this study did not assess the impact of the NCQs on student performance. To date, no study known to the authors has completed such an examination. If it were to be demonstrated that NCQs during RP exhibited negative implications on student performance on formal evaluations, future research should attempt to reduce these negative effects.

## Conclusions

This study investigated the implications of the implementation of RP in a large, undergraduate Gross Anatomy course. It was demonstrated that participation in RP was associated with improved student performance on formal evaluations. These findings showed that RP enhances long-term retention of semantic information, thereby improving learning, at least in Anatomical Education. This study has proven that the implementation of RP successfully provides positive implications in classroom settings, where strict experimental methods cannot ethically be used. In totality, teachers are encouraged to motivate their students to implement RP on their own. Furthermore, teachers are encouraged to implement testing, in the form of RP, in their classrooms ‘for’ learning in addition to the traditional testing ‘of’ learning in the form of formal evaluations.

## Data Availability

The datasets generated during and/or analyzed during the current study are available from the corresponding author on reasonable request.
